# Effectiveness of a multi-modal hospital-wide doctor mental health and wellness intervention

**DOI:** 10.1186/s12888-022-03908-0

**Published:** 2022-04-06

**Authors:** Katherine Petrie, Kelly Stanton, Aneesha Gill, Jennifer Simmons, Samuel B. Harvey

**Affiliations:** 1 Black Dog Institute, University of New South Wales, Randwick, NSW 2031 Australia; 2 School of Psychiatry, University of New South Wales, Sydney, NSW 2000 Australia; 3 St George Hospital, Kogarah, NSW 2217 Australia; 4 The Royal Brisbane and Women’s Hospital, Herston, Brisbane, QLD 4006 Australia; 5 Sutherland Hospital, Caringbah, NSW 2229 Australia; 6 University of New South Wales, Sydney, NSW 2000 Australia

**Keywords:** Doctor, Intervention, Common mental disorder, Workplace, Suicidal ideation

## Abstract

**Background:**

Doctors report high rates of workplace stress and are at increased risk of mental health disorders. However, there are few real-world studies evaluating the effectiveness of interventions aimed at addressing workplace risk factors and improving doctors’ mental health in a hospital setting. This study was conducted over two years (2017–2019) to assess the effects of a multi-modal intervention on working conditions doctors’ mental health and help-seeking for mental health problems in two Australian teaching hospitals.

**Methods:**

The multimodal intervention consisted of organisational changes, such as reducing unrostered overtime, as well as strategies for individual doctors, such as mental health training programs. Hospital-based doctors at all career stages were eligible to participate in two cross-sectional surveys. 279 doctors completed the baseline survey (19.2% response rate) and 344 doctors completed the follow-up survey (31.3% response rate). A range of workplace risk and protective factors, mental health (psychological distress and suicidal ideation) and help-seeking outcomes were assessed.

**Results:**

There were significant improvements in key workplace protective factors, with small effects found for doctors’ job satisfaction, stress, work-life balance and perceived workplace support and a significant reduction in workplace risk factors including a moderate reduction in reported bullying behaviour between baseline to follow-up (job satisfaction *p* < 0.05, all other outcomes *p* < 0.01). However, no significant changes in doctors’ mental health or help-seeking outcomes were found over the intervention period.

**Conclusion:**

Following the implementation of individual and organisational-level strategies in two Australian tertiary hospitals, doctors reported a reduction in some key workplace stressors, but no significant changes to their mental health or help-seeking for mental health problems. Further research is warranted, particularly to determine if these workplace changes will lead to improved mental health outcomes for doctors once maintained for a longer period.

**Supplementary Information:**

The online version contains supplementary material available at 10.1186/s12888-022-03908-0.

## Background

International evidence indicates that doctors experience elevated rates of occupational stress, common mental disorders and suicide compared to other workforces [1–4]. Concerningly, international [2, 4] and Australian data [5, 6] identifies particularly high rates of depression and suicidal ideation, and low rates of help-seeking among doctors-in-training. There have been increasing calls for hospitals and health systems to take active measures to better protect the mental health and wellbeing of their medical staff. The need for such measures has become even greater given the COVID-19 pandemic [7]. However, to date it remains unknown if hospital-based interventions can improve the mental health of doctors due to a lack of controlled studies evaluating organisational-level interventions among medical professionals [8].

Poor mental health among doctors has been associated with a range of adverse workplace factors, including long work hours, high job demands, and work-life imbalance [4, 9, 10]. A supportive workplace can help protect against employee mental ill-health [11] with emerging evidence that supervisor behaviour is an important predictor of mental health among employees, including health professionals [12, 13]. Broader issues within medicine, including stigma and concerns around possible reporting to the health regulators [14, 15] likely also contribute to low rates of help-seeking among doctors [5, 16].

Current best-practice models of workplace mental health now recognise that employee wellbeing is multifactorial and requires interventions that simultaneously address individual, team, organisational and systemic factors [17, 18]. Evidence suggests that these multi-modal approaches are more effective than a single intervention [19, 20]. The importance of providing both individual and organisational or structural solutions is now well-established for creating more mentally healthy workplaces [20, 21]. Despite this, there has been very limited research published to date evaluating interventions to reduce psychological distress or suicidal ideation and improve workplace conditions for doctors, whether using single or multiple strategies. A systematic review and meta-analysis identified only eight controlled trials of individual-level interventions, and no controlled studies of organisational-level interventions to reduce common mental disorders among doctors [8]. What is lacking are published evaluations of the effectiveness of hospital-based mental health interventions amongst doctors that have been undertaken in a real-world setting.

This study aimed to assess the effects of a multi-modal intervention on workplace factors and doctors’ mental health in two Australian hospitals. The study addressed four primary research questions. Does a hospital wide multi-modal intervention: 1) shift workplace risk factors known to be important in doctor wellbeing; 2) reduce mental health symptoms, specifically psychological distress and suicidal ideation among doctors; 3) impact doctors’ help-seeking attitudes or behaviour for mental health problems; and 4) is the intervention feasible and acceptable?. Given that different work stressors are likely to be more salient for doctors at different stages of their career, a secondary research question examined whether the intervention had varying impact on different groups of doctors.

## Methods

### Study design, setting and sample

The study was conducted over a two-year period (2017–2019) in two public teaching hospitals within the same metropolitan Local Health District in New South Wales, Australia. All doctors employed full-time at that timepoint in either hospital were eligible to participate in each survey, and eligible participants could participate in one or both of the surveys. All eligible participants were invited to participate in each survey. This strategy was justified on practical grounds given the often transient nature of the medical workforce, particularly where medical training programs requiring junior doctors to move from hospital to hospital throughout their training. The total sample size eligible for inclusion was 1,455 individuals at baseline (2017) and 1,110 at follow-up (2019).

### Ethics approval

This project was assessed by the South Eastern Sydney Local Health District Human Research Ethics Committee who determined that this project could be undertaken as a quality improvement and quality assurance project not requiring independent ethics review, under the NSW Health Guideline *GL2007_020 Human Research Ethics Committees – Quality Improvement and Ethics Review: A Practice Guide for NSW.* All methods were carried out in accordance with these guidelines and regulations.

### Procedure

All eligible participants were sent an email at baseline (September 2017) and follow-up (September 2019) containing information about the study, an invitation to participate, and a unique single-use URL link to the online questionnaire. Participants were advised that this was an independent research study and all responses were confidential and anonymous. Each survey remained open for approximately four weeks. Up to three reminder emails were circulated by hospital administration staff to all participants. All participants provided informed consent at each survey.

#### Intervention

**Fig. 1:**
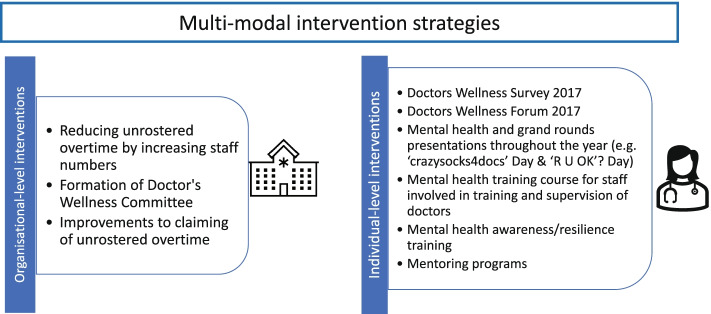
Strategies implemented under the multi-modal intervention

The multi-modal intervention consisted of evidence-informed strategies directed at an individual-level to each doctor, either individually or in a group, and at an organisational-level to change workplace practice and culture (Fig. 1). Nine strategies were selected on the basis of existing evidence [8, 19, 22, 23] and informed by the results of the baseline survey that indicated areas for improvement within the workplace.

#### Organisational-level interventions

- Strategy 1: Reducing unrostered overtime by increasing staff numbers – employment of 1 additional full-time junior doctor.
- Strategy 2: Formation of Doctors Wellness Committee – 5 meetings per year.
- Strategy 3: Improvements to claiming of unrostered overtime – improvements to process, including removal of pre-approval requirement and streamlining of online claiming process, and ensuring staff awareness of process by increased communication and promotion.

#### Individual-level interventions

- Strategy 4: Doctors Wellness Survey 2017
- Strategy 5: Doctors Wellness Forum 2017 – to communicate results of survey and to discuss next steps; one-off; 3 h; optional attendance; participation: *n* > 100 doctors.
- Strategy 6: Mental health and grand rounds presentations throughout the year – 2 per year; 1 h; mandatory attendance at grand rounds; participation: *n* > 100 doctors.
- Strategy 7: Mental health training course for all staff involved in training and supervision of doctors –3 h; same session offered twice; optional attendance; participation: *n* = 50 staff.
- Strategy 8: Mental health awareness/resilience training – 1 session; 2 h; doctors-in-training; mandatory.
- Strategy 9: Mentoring programs – informal peer support ‘buddy’ program for interns; at least one term (10 weeks); optional participation.

The intervention programs were implemented over a period of 18 months between December 2017 to August 2019 with endorsement from hospital executive and driven by the Hospital Doctors Wellness Committee. Programs were advertised internally in the hospital, during doctors’ education sessions and at least once by email to all doctors. Doctors were able to participate in as many strategies as they were able, and all strategies were implemented in working hours.

### Measures

The baseline survey contained 57 items, and the follow-up survey contained 73 items. 55 items were retained in the follow-up survey. The majority of measures were sourced from validated, standardised instruments or based on items from Australian large-scale surveys of doctors (Beyondblue National Mental Health Survey of Doctors and Medical Students, October 2013 [6]; the Australian Medical Association (AMA) Junior Doctors Survey (2007) [24]; Medicine in Australia: Balancing Employment and Life (MABEL) Survey [25, 26] with some tailored items assessing hospital and intervention-specific outcomes.

#### Demographics

Demographic items included age, gender, hospital site, area of specialty, stage of career, presence of children under 18 years at home, and in the follow-up survey, whether they had participated in the baseline survey.

#### Workplace variables

A number of workplace variables were examined using tailored and standardised measures; working hours in the past week, job satisfaction, global work-related stress, work-life balance, workplace support, workload, and bullying and harassment.

Hours worked in the past week (rostered): numeric (0–168 h).

Overall job satisfaction as a single tailored item: ‘*How would you rate your overall level of job satisfaction with your current role?*’; “extremely satisfied” (5) to “extremely unsatisfied” (1) (range: 1–5) based broadly on items in the MABEL survey [26]. Higher scores indicated greater overall job satisfaction.

A list of 21 work-related stressors were assessed using an item from the Australian Beyondblue National Mental Health Survey of Doctors and Medical Students survey [6]. Participants indicated the degree to which they had been stressed by each work-related event; “not at all stressed” (0), “somewhat stressed (1), “very stressed” (2). Items included e.g. “*difficult relations with senior colleagues”, “fear of making mistakes”, “litigation fears*”. An overall stress score summed across all stressors was created (range: 0–42), where higher scores indicated a higher degree of overall work-related stress.

Perceived level of work-life balance over the past 12 months was assessed using a single self-devised item: ‘*I feel I have a good work-life balance*’. Participants were asked to rate their agreement with the statement; “strongly disagree” (1) – “strongly agree” (5), where higher scores indicated a better perceived work-life balance over the past year (range: 1–5).

Workplace support over the past 12 months was assessed using one self-devised item. Participants were asked to indicate their agreement to the following statement: ‘*I have found my hospital administration to be helpful/supportive*’; “strongly disagree” (1) – “strongly agree” (5), where higher scores indicated a higher level of perceived support in the workplace (range: 1–5).

Participants were asked to assess their workload over the last 12 months, by indicating their agreement to a single tailored item based on the MABEL survey [26]: ‘*I consider that my workload has been excessive’*; “strongly disagree” (1) – “strongly agree” (5), where higher scores indicated a more excessive workload (range: 1–5).

Workplace bullying and harassment were assessed by a single tailored item based on the Beyondblue survey [6]. Participants were asked to indicate their level of agreement with the following statement in relation to the past 12 months: ‘*I have experienced or witnessed bullying in the workplace’*; “strongly disagree” (1) – “strongly agree” (5). Higher scores indicated more exposure to bullying in the workplace in the past year.

#### Mental health outcomes

Two mental health outcomes were assessed: psychological distress and suicidal ideation.

Psychological distress was assessed using the 10-item Kessler Psychological Distress Scale (K10) [27], a widely used standardised screening tool for mental illness validated in the general population with consistent psychometric properties [28] and considered comparable to clinician-based assessment.

Suicidal ideation was assessed by a single yes/no item: ‘*During the last 12 months have you had thoughts of taking your own life or of deliberately hurting yourself?*’, based on the item used in the Beyondblue survey [6] and on the item used in the 2007 ABS National Survey of Mental Health and Wellbeing conducted on a representative sample of the Australian adult population [29].

#### Help-seeking outcomes

Help-seeking for mental health problems was assessed by two items with yes/no responses; i) confidence around help-seeking: ‘*Whether or not you have been diagnosed with or are experiencing mental health problems, would you feel confident or comfortable seeking help for these problems?*’; ii) actual help-seeking behaviours in the last year ‘*In the last 12 months, have you sought support or treatment for any mental health problems?*’, based on the items used in the Beyondblue survey [6].

### Statistical analyses

Standardised mean differences (SMD), calculated manually as Cohen’s d [30], were used to compare the scores of pre and post intervention groups on main outcome measures. Regression models were used to test of differences between baseline and follow-up surveys in terms of key workplace risk factors and mental health and help-seeking outcomes. The adjusted models accounted for any demographic differences between the baseline and follow-up samples. Linear regression was conducted for psychological distress whilst logistic regression was conducted for suicidal ideation. The regression models were repeated in three separate groups; i) interns; ii) residents and registrars; iii) consultants and fellows in planned sensitivity analyses. Adjusted *p* values are reported for all comparisons between baseline and follow-up surveys. All analyses were undertaken in IBM SPSS (v25) and STATA (v12).

## Results

There were 1,455 doctors employed across the two hospital sites in 2017. It is not clear how many of these viewed the emailed invitation, although 367 participants started and 279 completed the survey (19.2% response rate). At follow-up 1,110 doctors were invited to participate, of whom 416 started and 344 fully completed the survey (31.3% response rate, 344/1110). Response rates were significantly different between timepoints (Chi^2^ = 28.3; *p* < 0.01). In keeping with the transient nature of many training medical positions, the majority of the 2019 sample did not participate in the 2017 survey (72.2%, 216/299). Despite this, the two samples were generally similar in terms of demographic and career characteristics (Table 1). There was a significant difference between timepoints in terms of the type of medical degree undertaken (undergraduate vs postgraduate) and whether the doctors had children living at home. As a result, these two demographics were controlled for in subsequent regression models.

**Table 1:** Participant characteristics at baseline and follow-up

	**Baseline (2017 sample)**	**Follow-up (2019 sample)**	***p* value**^a^
	**n (%)**	**n (%)**	
**Age**	*N* = 325	*N* = 322	0.63
21 – 30 years	150 (46.2)	142 (44.1)	
31 – 40 years	96 (29.5)	86 (26.7)	
41 – 50 years	37 (11.4)	45 (14.0)	
51 – 60 years	29 (8.9)	33 (10.2)	
> 61 years	13 (4.0)	16 (5.0)	
**Gender**	*N* = 324	*N* = 322	0.87
Male	171 (52.8)	172 (53.4)	
Female	153 (47.2)	150 (46.6)	
Type of medical degree**	*N* = 327	N = 322	0.001
Undergraduate	270 (82.6)	190 (59.0)	
Postgraduate	57 (17.4)	132 (41.0)	
**Relationship**	*N* = 327	*N* = 322	0.69
Single, never married	79 (24.2)	66 (20.5)	
In a committed relationship	98 (30.0)	96 (29.8)	
Married	141 (43.1)	149 (46.3)	
Separated/divorced/widowed	< 10 (2.7)	11 (3.4)	
Children under 18*	*N* = 327	*N* = 322	0.04
Yes	66 (20.2)	87 (27.0)	
No	261 (79.8)	235 (73.0)	
**Hospital**	*N* = 320	*N* = 310	0.92
Site 1	273 (85.3)	261 (84.2)	
Site 2	37 (11.6)	38 (12.3)	
**Stage of career**	*N* = 309	*N* = 299	0.68
Interns^1^	55 (17.8)	50 (16.7)	
Residents^2^	58 (18.8)	60 (20.1)	
Registrars^3^	115 (37.2)	100 (33.4)	
Consultants/Fellows	81 (26.2)	89 (29.8)	
Area of specialty*	*N* = 291	*N* = 297	0.023
Anaesthetics	28 (9.6)	16 (5.0)	
Emergency medicine	56 (19.2)	41 (14.0)	
ICU/critical care	20 (6.9)	23 (7.7)	
Physician subspecialties^4^	108 (37.1)	138 (46.5)	
Psychiatry	< 10 (2.7)	15 (5.1)	
Obstetrics and gynaecology	< 10 (1.7)	13 (4.4)	
Surgical specialties^5^	34 (11.7)	25 (8.4)	
JMOs^6^	29 (10.0)	25 (8.4)	
Other^7^	< 10 (1.0)	< 10 (0.3)	

NB: rows less than 10 are shown as < 10 to maintain anonymity

^*^p < 0.05

^**^p < 0.01

^a^2 sided *p* value for Chi^2^ test

^1^includes Interns

^2^includes residents and Junior Medical Officers

^3^includes registrars and unaccredited registrars

^4^includes specialties listed under the Royal Australasian College of Physicians including Basic and Advanced Physician Trainees, Geriatric medicine, Cardiology, Gastroenterology, Endocrinology, Medical oncology, Haematology, General medicine, Infectious Diseases, Immunology, Nephrology, Neurology, Nuclear Medicine, Respiratory and Sleep medicine, Rheumatology, Palliative medicine, Sexual health, Addiction medicine, Rehabilitation medicine, Paediatric medicine

^5^includes surgical specialties listed under the Royal Australasian College of Surgeons: General surgery, Breast/Endocrine, Otolaryngology Head and Neck surgery, Neurosurgery, Upper Gastrointesitial, Trauma, Liver, Colorectal, Vascular, Cardiothoracics, Urology, Plastic and reconstructive surgery, Orthopaedic surgery

^6^includes responses of intern, JMO, RMO, SRMO, relief, PGY1, shift work, night shifts

^7^includes dermatology and radiology

There was a statistically significant difference between total K10 scores of doctors at different career stages (F(1,486) = 14.54, *p* < 0.01). A comparison of means F(3,484) = 16.33, *p* < 0.001) showed that the mean K10 score for consultants and fellows was lower than the scores for the other groups. The proportion of doctors reporting suicidal ideation was not statistically significantly different between the groups (Chi^2^ = 4.33, df = 3, *p* = 0.228).

There was a significant improvement in key workplace protective factors following the intervention, with increased job satisfaction (mean difference in scores = 0.26; *p* < 0.05), feeling supported by administration (mean difference in scores = 0.4; *p* < 0.01) and work-life balance (mean difference in scores = 0.4; *p* < 0.01) post intervention (Table 2). There was also a significant reduction in workplace risk factors including bullying (mean difference in scores = -0.5; *p* < 0.01), excessive workload (mean difference in scores = -0.2; *p* < 0.01) and overall stress (mean difference in scores = -2.0; *p* < 0.01). Sensitivity analyses (Additional Tables 1, 2, 3, 4, 5 and 6) found that for the consultants/fellows and for the interns, there were no significant differences in workplace factors following the intervention with the exception of an increase in perceived support from hospital administration among interns (*p* = 0.001). However, among registrars and residents, significant improvements were seen in several workplace risk factors (*p* < 0.001 and *p* < 0.05) in the adjusted analyses, namely perceived administrative support (mean difference in scores = 0.39; *p* = 0.023), work-life balance (mean difference in scores = 0.41; *p* = 0.001), excessive workload (mean difference in scores = -0.44; *p* < 0.001) and bullying (mean difference in scores = -0.44; *p* = 0.032). (Additional Table 3).

**Table 2:** The effect of a multi-modal doctor intervention on workplace factors (unadjusted and adjusted analyses). Mean (SD) values for each risk factor are shown before and after the intervention, with standardised mean differences (SMD) used to allow comparison of the effect sizes

	**Unadjusted**			**Adjusted**^b^
	**Baseline (2017 sample)**	**Follow-up (2019 sample)**		
	**Mean (SD); range**	**Mean (SD); range**	**SMD**^a^	***p* value**
Hours worked/week	46.3 (17.5); 0–160	45.9 (13.9); 0–100	0.025	.708
Job satisfaction	3.5 (.99)	3.76 (.98)	-0.26	.035
Overall stress	14.0 (6.5)	12.0 (6.0)	0.32	.001
Support (administration)	3.0 (1.1)	3.4 (1.3)	-0.34	.004
Work-life balance	2.6 (1.0)	3.0 (1.1)	-0.37	.001
Excessive workload	3.5 (.9)	3.3 (1.1)	0.20	.029
Bullying	3.5 (1.2)	3.0 (1.3)	0.40	< .001

^a^Standardised Mean Difference

^b^Adjusted for type of medical degree and presence of children at home

There were no significant changes in the K10 score (mean difference in scores = 0.90, *p* = 0.12) or the proportion of doctors reporting suicidal ideation following the intervention (Table 3). There were high levels of reported suicidal ideation in the last 12 months at baseline (11.8%, 26/220). Post-intervention there was a decrease in suicidal ideation to 8.5% (20/236), however this did not reach statistical significance. Rates remained similar to previously reported national averages for doctors [6]. There was no significant difference in doctors’ level of reported confidence in seeking help for mental health problems or in their actual help-seeking behaviours following the intervention. A similar pattern of results was seen when each group of doctors (interns, residents/registrars, consultants/fellows) were examined separately in sensitivity analyses (all *p* > 0.05) (Additional Tables 1, 2, 3, 4, 5 and 6).

**Table 3:** Comparisons of mental health and help-seeking outcomes (unadjusted and adjusted) before and after a multi-modal doctor intervention

	**Unadjusted**			**Adjusted**^a^
	**Baseline (2017 sample)**	**Follow-up (2019 sample)**		
	**Mean (SD); range**	**Mean (SD); range**	***p* value**	***p* value**
Psychological distress	18.6 (6.3); 10—50	17.7 (6.5); 10—50	.12	.203
	**n (%) Yes**	**n (%) Yes**		
Suicidal ideation	26 (11.8)	20 (7.8)	.162	.182
Help-seeking confidence	131 (58.2)	157 (61.3)	.515	.376
Help-seeking behaviour	38 (17.3)	33 (12.9)	.198	.511

^a^Adjusted for type of medical degree and presence of children at home

Almost half of the 2019 sample of doctors (48%; 200/416) were aware of three or more of the intervention programs. A majority of doctors (65.2%; 227/348) felt that the programs had had a positive impact on the culture around mental health within their hospital whilst 44.5% (155/348) reported a positive impact on their own well-being.

## Discussion

This is one of the first published studies to evaluate the real-world effectiveness of a multi-modal doctor wellness intervention targeting both organisational and individual factors in a sample of employed medical practitioners. This study demonstrated that a range of strategies can be implemented in the real-world metropolitan hospital setting with mixed effects. The intervention was successful in enhancing protective factors, namely job satisfaction, feeling supported by administration and work-life balance and decreasing several workplace risk factors, including overall stress, excessive workload, and bullying. However, despite this, there were no significant changes in self-reported psychological distress and suicidal ideation in the overall sample.

Comparing our results with similar research is difficult as comparable studies with doctors are limited, however some positive effects on physician burnout have been identified, especially for organisational-level interventions in a review of controlled studies [22]. Similar hospital-based organisational interventions among healthcare professionals have also yielded promising effects on work-based risk and protective factors [31] which are consistent with our findings, as well as mental health outcomes [23, 32]. The nine strategies that made up this multi-modal intervention were selected following appraisal of the available evidence, feedback from medical staff and consideration on what was practical to be achieved within usual operational constraints and without any additional funding. Importantly, this study shows that not only is this type of coordinated multi-modal intervention feasible, but there is also evidence of real-world measurable improvements in doctors’ job satisfaction, stress, work-life balance, perceived support and a reduction in reported bullying behaviour. The results demonstrate that this type of hospital-based multi-strategy intervention can generate significant benefits in several key workplace risk and protective factors across the medical workforce, with particular benefits for doctors-in-training. Each of these factors have been identified as key workplace risk factors for junior doctors’ mental ill-health [4, 9, 33]. As such, generating a meaningful reduction in each of these is an important outcome in itself.

However, the fact that these changes were not associated with a measurable improvement in doctors’ symptoms of psychological distress, suicidal ideation or help-seeking is notable and requires further consideration. A healthy worker effect [34] combined with type 2 error may play some part in this null finding for mental health outcomes. A larger sample of employed doctors may be needed in order to demonstrate the mental health protective effects of this change in risk factors. While the two-year time period of this study makes it longer in duration than most intervention trials in this population [8] many of the interventions were implemented over 18 months, meaning the expected changes in mental health outcomes may have taken longer than this period to manifest. Future studies could maintain these strategies over a longer period and evaluate their effects on mental health and workplace outcomes over a longer follow-up period, in line with the recognised need for more studies evaluating the longer-term effects of workplace mental health interventions among healthcare professionals [23, 35].

Sensitivity analyses showed that significant changes to workplace risk factors were seen among registrars and residents, but not among interns (except for an increase in perceived support from administration) or consultants and fellows, suggesting that the positive impacts of the intervention were concentrated among registrars and residents. The most likely reason for the difference in effectiveness is that many of the components of the intervention were focused on key issues that impact registrars and residents, such as unrostered overtime and mental health training for supervisors. Given that residents and registrars have consistently been shown to be at increased risk of mental ill-health compared to those later in their career [2, 4, 6, 36] and experience specific work and training-based stressors [9, 33], this finding suggests that that type of interventions used in this study are suitably targeted to this group. However, it also highlights that different interventions may need to be developed for doctors at other stages of their career and tailored by career stage.

The observed improvements in workplace outcomes may have been due to other factors in addition to or apart from the intervention strategies themselves, including the significant difference in specialties found between baseline and follow-up samples and the possibility that post-test completers were more likely to have been favorably impacted by the intervention than those who elected not to complete the survey. However, we adjusted for significant differences between samples in demographic variables and believe that this adequately controlled for one of the major potential confounds.

Issues around implementation and organisational feasibility are also important to consider. The strategies themselves are not without their own advantages and disadvantages, including requiring investment of staff time to implement on the ground, and for some, financial costs to be covered. However, a key focus of the intervention was implementing strategies that could be achieved during paid work hours with suitable cover arrangements for patients, and points to the need in such hospital-based programs for dedicated, protected time for medical staff for professional development, wellness, education and training, which is lacking in many hospitals. Importantly though, there was good support from the hospital executive that filtered down to the heads of department, and this managerical and executive support was critical and valuable in enhancing the implementation and the staff engagement with the strategies and in engineering top-down cultural change within the departments. Such high-level and employee support has been identified as a driver of implementation and positive effects of an intervention, especially for organisational-level strategies [21] and could help explain the observed improvements in workplace factors and culture.

Strengths of this study lie in its real-world evaluation of a multi-level intervention targeting and assessing both employee and workplace outcomes. However, there are limitations that need to be considered. As with most workplace surveys, particularly those involving doctor samples [6, 37], the response rate was relatively low and the survey included some non-validated and tailored and self-devised measures. However, we ensured all measures were based upon large-scale surveys with doctors or a representative Australian population, and so had face validity and were highly similar to existing national surveys. A key work-based barrier to doctors’ participation was the ability to attend sessions during work hours due to the high workload and competing demands of work. However, making some strategies mandatory and ensuring they were well-advertised increased participation. The sample may not be representative and there remains a risk of selection bias, although provided similar biases were present at both baseline and follow-up this alone should not invalidate our results. It is unclear if our results can be generalized to other countries or health systems. As anonymity was essential to encourage accurate self-reporting of mental health symptomatology [38], we were unable to link responses of returning participants across survey timepoints and cannot assess individual-level changes following the intervention. There are several limitations that affect the reliability and robustness of the findings and may have contributed to the null effects on mental health outcomes. These include the fact that baseline and follow-up data sets were neither independent samples nor featured a high proportion of respondents who completed both surveys. Whilst these issues do not prevent us from learning from the data, they are not unexpected in this type of research and understandable given the nature of the medical workforce and need to be taken into account when interpreting the findings. Both surveys had limitations in terms of response rate and representativeness, possibly due to anxieties many doctors have about discussing their own mental health, and the lack of a control group. It is impossible to determine which of the components of the multi-modal intervention generated the most change. All outcome measures were self-report, which are different to objective measures of the work environment and clinician-based diagnostic interviews. Finally, the strategies chosen as part of the multi-modal intervention tested in this study were specific to the hospitals involved. There are many other interventions that could have been included [8, 22], such as modifying work hours [39] or facilitated small-group physician discussion and learning programs [40] and in time it will be important for research to better define which interventions provide the most benefit. Notwithstanding these limitations, given that research regarding organisational interventions aimed at improving physicians’ mental health via modification of the work environment is urgently needed [8], our study remains a valuable addition to this literature and can inform and stimulate future studies.

## Conclusions

This study represents an early step in translating the emerging research around doctors’ mental health into improved wellbeing and mental health for doctors as well as improved working conditions within the health system. Following the implementation of individual and organisational-level strategies in two Australian tertiary hospitals, doctors reported a reduction in some key workplace stressors, but no significant changes to their mental health or help seeking. Further research is warranted, particularly to determine if these workplace changes will lead to improved mental health outcomes for doctors once maintained for a longer period.

## Supplementary Information

**Additional file 1:** **Table 1.** The effect of a multi-modal doctor intervention on workplace factors (unadjusted and adjusted analyses) on interns (n = 107).

**Additional file 2:** **Table 2.** Comparisons of mental health and help-seeking outcomes (unadjusted and adjusted) before and after a multi-modal doctor intervention among interns (n = 105).

**Additional file 3:** **Table 3.** The effect of a multi-modal doctor intervention on workplace factors (unadjusted and adjusted analyses) on residents and registrars (n = 333).

**Additional file 4:** **Table 4.** Comparisons of mental health and help-seeking outcomes (unadjusted and adjusted) before and after a multi-modal doctor intervention among residents and registrars (n = 333).

**Additional file 5:** **Table 5.** The effect of a multi-modal doctor intervention on workplace factors (unadjusted and adjusted analyses) on consultants and fellows (n = 170).

**Additional file 6:** **Table 6.** Comparisons of mental health and help-seeking outcomes (unadjusted and adjusted) before and after a multi-modal doctor intervention among consultants and fellows.

## Data Availability

The dataset generated and/or analysed during the current study are not publicly available to maintain the privacy of participants but are available from the corresponding author on reasonable request.

## Abbreviations

JMO: Junior Medical Officer
K10: Kessler Psychological Distress Scale 10-item
PGY1: Postgraduate Year 1
RMO: Resident Medical Officer
SD: Standard deviation
SMD: Standardised mean difference
SRMO: Senior Resident Medical Officer

## References

[1] Dutheil F, Aubert C, Pereira B, Dambrun M, Moustafa F, Mermillod M (2019). Suicide among physicians and health-care workers: A systematic review and meta-analysis. PloS One.

[2] Mata DA, Ramos MA, Bansal N, Khan R, Guille C, Di Angelantonio E (2015). Prevalence of depression and depressive symptoms among resident physicians: a systematic review and meta-analysis. JAMA.

[3] Ruitenburg MM, Frings-Dresen MHW, Sluiter JK (2012). The prevalence of common mental disorders among hospital physicians and their association with self-reported work ability: a cross-sectional study. BMC Health Serv Res.

[4] Tyssen R, Vaglum P (2002). Mental health problems among young doctors: an updated review of prospective studies. Harv Rev Psychiatry.

[5] Muhamad Ramzi NSA, Deady M, Petrie K, Crawford J, Harvey S. Help‐seeking for depression among Australian doctors. Int Med J. 2020;51(12):2069–77. 10.1111/imj.15035 32833296

[6] Wu F, Ireland M, Hafekost K, Lawrence D. National mental health survey of doctors and medical students. Beyondblue. 2013. Available at: http://www.beyondblue.org.au/docs/default-source/default-document-library/bl1132-report---nmhdmss-full-report_web.pdf.

[7] Gold JA (2020). Covid-19: adverse mental health outcomes for healthcare workers. BMJ.

[8] Petrie K, Crawford J, Baker ST, Dean K, Robinson J, Veness BG (2019). Interventions to reduce symptoms of common mental disorders and suicidal ideation in physicians: a systematic review and meta-analysis. Lancet Psychiatry.

[9] Petrie K, Crawford J, Shand F, Harvey SB (2021). Workplace stress, common mental disorder and suicidal ideation in junior doctors. Intern Med J.

[10] Tyssen R, Vaglum P, Grønvold NT, Ekeberg Ø (2000). The impact of job stress and working conditions on mental health problems among junior house officers. A nationwide Norwegian prospective cohort study. Medical education.

[11] Modini M, Joyce S, Mykletun A, Christensen H, Bryant RA, Mitchell PB (2016). The mental health benefits of employment: results of a systematic meta-review. Australas Psychiatry.

[12] Gayed A, Kugenthiran N, LaMontagne AD, Christensen H, Glozier N, Harvey SB (2021). Can an online mental health training program improve physician supervisors’ behaviour towards trainees?. Int Med J.

[13] Petrie K, Gayed A, Bryan BT, Deady M, Madan I, Savic A (2018). The importance of manager support for the mental health and well-being of ambulance personnel. PloS One.

[14] AHPRA. Making a mandatory notification 2020 [Available from: https://www.ahpra.gov.au/Notifications/mandatorynotifications/Mandatory-notifications.aspx.

[15] Guille C, Speller H, Laff R, Epperson CN, Sen S (2010). Utilization and barriers to mental health services among depressed medical interns: a prospective multisite study. J Grad Med Educ.

[16] Dyrbye LN, West CP, Sinsky CA, Goeders LE, Satele DV, Shanafelt TD (2017). Medical licensure questions and physician reluctance to seek care for mental health conditions. Mayo Clin Proc.

[17] Henderson M, Harvey SB, Øverland S, Mykletun A, Hotopf M (2011). Work and common psychiatric disorders. J R Soc Med.

[18] Joyce S, Modini M, Christensen H, Mykletun A, Bryant R, Mitchell PB (2016). Workplace interventions for common mental disorders: a systematic meta-review. Psychol Med.

[19] Foundation BOHR (2005). Workplace interventions for people with common mental health problems: Evidence review and recommendations.

[20] Petrie K, Joyce S, Tan L, Henderson M, Johnson A, Nguyen H (2018). A framework to create more mentally healthy workplaces: a viewpoint. Aust N Z J Psychiatry.

[21] Harvey SB, Joyce S, Tan L, Johnson A, Nguyen H, Modini M, et al. Developing a mentally healthy workplace: A review of the literature. Australia: Black Dog Institute; 2014. Available at: https://www.headsup.org.au/docs/default-source/resources/developing-a-mentally-healthy-workplace_final-november-2014.pdf?sfvrsn=8.

[22] Panagioti M, Panagopoulou E, Bower P, Lewith G, Kontopantelis E, Chew-Graham C (2017). Controlled interventions to reduce burnout in physicians: a systematic review and meta-analysis. JAMA Intern Med.

[23] Ruotsalainen J, Serra C, Marine A, Verbeek J (2008). Systematic review of interventions for reducing occupational stress in health care workers. Scand J Work Environ Health.

[24] Australian Medical Association. AMA Survey Report on Junior Doctor Health and Wellbeing. Australian Capital Territory: Australian Medical Association Ltd; 2008. Available at https://ama.com.au/sites/default/files/documents/JDHS_report_FINAL_0.pdf.

[25] Joyce CM, Scott A, Jeon S-H, Humphreys J, Kalb G, Witt J, et al. The" Medicine in Australia: Balancing Employment and Life (MABEL)" longitudinal survey-protocol and baseline data for a prospective cohort study of Australian doctors’ workforce participation. BMC Health Serv Res. 2010;10(1):1–10. 10.1186/1472-6963-10-50 PMC2837653 20181288

[26] Szawlowski S, Taylor T, Scott A, Leahy A. Mabel user manual: Wave 10 release. Victoria: Melbourne Institute of Applied Economic and Social Research The University of Melbourne; 2019. Available at: https://melbourneinstitute.unimelb.edu.au/__data/assets/pdf_file/0008/2956463/MABEL-User-Manual-Wave-10_Jan2019.pdf.

[27] Kessler RC, Andrews G, Colpe LJ, Hiripi E, Mroczek DK, Normand SLT (2002). Short screening scales to monitor population prevalences and trends in non-specific psychological distress. Psychol Med.

[28] Andrews G, Slade T (2001). Interpreting scores on the Kessler psychological distress scale (K10). Aust N Z J Public Health.

[29] Statistics ABo (2009). 2007 National Survey of Mental Health and Wellbeing: users’ guide (cat. no. 4327).

[30] Cohen J (1992). Statistical power analysis. Curr Dir Psychol Sci.

[31] Bourbonnais R, Brisson C, Vézina M (2011). Long-term effects of an intervention on psychosocial work factors among healthcare professionals in a hospital setting. Occup Environ Med.

[32] Bourbonnais R, Brisson C, Vinet A, Vezina M, Abdous B, Gaudet M (2006). Effectiveness of a participative intervention on psychosocial work factors to prevent mental health problems in a hospital setting. Occup Environ Med.

[33] Zhou AY, Panagioti M, Esmail A, Van Tongeren M, Bower P. Factors associated with burnout and stress in trainee physicians: a systematic review and meta-analysis. JAMA Netw Open. 2020;3(8):e2013761-e. 10.1001/jamanetworkopen.2020.13761 PMC7435345 32809031

[34] Chowdhury R, Shah D, Payal AR (2017). Healthy worker effect phenomenon: revisited with emphasis on statistical methods–a review. Indian J Occup Environ Med.

[35] Gray P, Senabe S, Naicker N, Kgalamono S, Yassi A, Spiegel JM (2019). Workplace-based organizational interventions promoting mental health and happiness among healthcare workers: A realist review. Int J Environ Res Public Health.

[36] Wijeratne C, Johnco C, Draper B, Earl JK (2021). Older physicians’ reporting of psychological distress, alcohol use, burnout and workplace stressors. Am J Geriatr Psychiatry.

[37] Bonevski B, Magin P, Horton G, Foster M, Girgis A (2011). Response rates in GP surveys: trialling two recruitment strategies. Aust Fam Physician.

[38] Marshall RE, Milligan-Saville J, Petrie K, Bryant RA, Mitchell PB, Harvey SB (2021). Mental health screening amongst police officers: factors associated with under-reporting of symptoms. BMC Psychiatry.

[39] Ali NA, Wolf KM, Hammersley J, Hoffmann SP, O’Brien JM Jr, Phillips GS, et al. Continuity of care in intensive care units: a cluster-randomized trial of intensivist staffing. Am J Respir Crit Care Med. 2011;184(7):803–8. 10.1164/rccm.201103-0555OC 21719756

[40] West CP, Dyrbye LN, Rabatin JT, Call TG, Davidson JH, Multari A (2014). Intervention to promote physician well-being, job satisfaction, and professionalism: a randomized clinical trial. JAMA Intern Med.

