# Supplementary material for: Effectiveness of a multi-modal hospital-wide doctor mental health and wellness intervention
Source: BMC Psychiatry. 2022 Apr 6;22:244. doi: 10.1186/s12888-022-03908-0 (PMC8983801; doi:10.1186/s12888-022-03908-0)
Supplement: Supplementary file 1 — Additional file 1: Table 1. The effect of a multi-modal doctor intervention on workplace factors (unadjusted and adjusted analyses) on interns (n = 107). [file 12888_2022_3908_MOESM1_ESM.docx]

**Additional Table 1.** The effect of a multi-modal doctor intervention on workplace factors (unadjusted and adjusted analyses) on interns (n = 107).

Mean (SD) values for each risk factor are shown before and after the intervention, with standardised mean differences (SMD) used to allow comparison of the effect sizes.

|  | **Unadjusted** | |  | |  | | **Adjusted^$^** |
| --- | --- | --- | --- | --- | --- | --- | --- |
|  | **Baseline (2017 sample)** | **Follow-up (2019 sample)** |  | |  | |  |
|  | **Mean (SD); min - max** | **Mean (SD); min - max** | | **SMD^%^** | | **p value** | **p value** |
| Hours worked/week | 44.11 (12.45), 0-70 | 45.63 (14.9); 0-85 | -1.52 | | 0.58 | | 0.07 |
| Job satisfaction | 3.34 (0.96) | 3.83 (0.93) |  | |  | | 0.09 |
| Overall stress | 14.5 (6.39); 3-27 | 12.32 (4.44); 4-22 | 2.18 | | 0.10 | | 0.28 |
| Support (administration) | 3.19 (0.80) | 3.93 (0.96) | -0.74 | | <0.001 | | 0.001 |
| Work-life balance | 2.82 (0.95) | 3.24 (1.03) | -0.43 | | 0.045 | | 0.22 |
| Excessive workload | 3..30 (0.95) | 3.40 (0.94) | -0.11 | | 0.60 | | 0.92 |
| Bullying | 3.84 (1.08) | 3.56 (1.08) | 0.29 | | 0.22 | | 0.36 |

^$^ Adjusted for type of medical degree and presence of children at home.

^%^ Standardised Mean Difference
