# Supplementary material for: Effectiveness of a multi-modal hospital-wide doctor mental health and wellness intervention
Source: BMC Psychiatry. 2022 Apr 6;22:244. doi: 10.1186/s12888-022-03908-0 (PMC8983801; doi:10.1186/s12888-022-03908-0)
Supplement: Supplementary file 2 — Additional file 2: Table 2. Comparisons of mental health and help-seeking outcomes (unadjusted and adjusted) before and after a multi-modal doctor intervention among interns (n = 105). [file 12888_2022_3908_MOESM2_ESM.docx]

**Additional Table 2.** Comparisons of mental health and help-seeking outcomes (unadjusted and adjusted) before and after a multi-modal doctor intervention among interns (n = 105).

|  | **Unadjusted** | | | |  |  | | **Adjusted^$^** |
| --- | --- | --- | --- | --- | --- | --- | --- | --- |
|  | | **Baseline (2017 sample)** | | **Follow-up (2019 sample)** |  |  | |  |
|  | | **Mean (SD); min - max** | **Mean (SD); min - max** | | **SMD^%^** | **p value** | | **p value** |
| Psychological distress | | 18.49 (6.14); 11-33 | | 45.63 (14.91); 0-95 | -0.16 | 0.90 | | 0.60 |
|  | | **n (% within year of data collection) Yes** | | **n (% within year of data collection) Yes** |  |  | |  |
| Suicidal ideation | | 7 (17.5) | | 6 (13.6) |  | 0.63 | | 0.55 |
| Help-seeking confidence | | 26 (63.4) | | 27 (61.4) |  | 0.85 | 0.65 | |
| Help-seeking behaviour | | 9 (22.0) | | 9 (20.5) |  | 0.87 | 0.47 | |

^$^ Adjusted for type of medical degree and presence of children at home.

^%^ Standardised Mean Difference
