# Supplementary material for: Effectiveness of a multi-modal hospital-wide doctor mental health and wellness intervention
Source: BMC Psychiatry. 2022 Apr 6;22:244. doi: 10.1186/s12888-022-03908-0 (PMC8983801; doi:10.1186/s12888-022-03908-0)
Supplement: Supplementary file 3 — Additional file 3: Table 3. The effect of a multi-modal doctor intervention on workplace factors (unadjusted and adjusted analyses) on residents and registrars (n = 333). [file 12888_2022_3908_MOESM3_ESM.docx]

**Additional Table 3.** The effect of a multi-modal doctor intervention on workplace factors (unadjusted and adjusted analyses) on residents and registrars (n = 333).

Mean (SD) values for each risk factor are shown before and after the intervention, with standardised mean differences (SMD) used to allow comparison of the effect sizes.

|  | **Unadjusted** | |  |  | **Adjusted^$^** |
| --- | --- | --- | --- | --- | --- |
|  | **Baseline (2017 sample)** | **Follow-up (2019 sample)** |  |  |  |
|  | **Mean (SD); min - max** | **Mean (SD); min - max** | **SMD^%^** | **p value** | **p value** |
| Hours worked/week | 47.73 (15.3) | 46.57 (13.8) | 1.16 | 0.48 | 0.19 |
| Job satisfaction | 3.56 (0.94) | 3.61 (1.03) | -0.05 | 0.67 | 0.09 |
| Overall stress | 15.0 (5.87) | 13.14 (6.38) | 1.87 | 0.02 | 0.12 |
| Support (administration) | 3.10 (1.10) | 3.49 (1.18) | -0.39 | 0.005 | 0.023 |
| Work-life balance | 2.42 (0.97) | 2.83 (1.11) | -0.41 | 0.001 | <0.001 |
| Excessive workload | 3.76 (0.86) | 3.32 (1.11) | 0.43 | <0.001 | 0.001 |
| Bullying | 3.50 (1.22) | 3.06 (0.94) | 0.44 | 0.004 | 0.032 |

^$^ Adjusted for type of medical degree and presence of children at home.

^%^ Standardised Mean Difference
