# Supplementary material for: Effectiveness of a multi-modal hospital-wide doctor mental health and wellness intervention
Source: BMC Psychiatry. 2022 Apr 6;22:244. doi: 10.1186/s12888-022-03908-0 (PMC8983801; doi:10.1186/s12888-022-03908-0)
Supplement: Supplementary file 4 — Additional file 4: Table 4. Comparisons of mental health and help-seeking outcomes (unadjusted and adjusted) before and after a multi-modal doctor intervention among residents and registrars (n = 333). [file 12888_2022_3908_MOESM4_ESM.docx]

**Additional Table 4.** Comparisons of mental health and help-seeking outcomes (unadjusted and adjusted) before and after a multi-modal doctor intervention among residents and registrars (n = 333).

|  | **Unadjusted** | | | |  |  | | **Adjusted^$^** |
| --- | --- | --- | --- | --- | --- | --- | --- | --- |
|  | | **Baseline (2017 sample)** | | **Follow-up (2019 sample)** |  |  | |  |
|  | | **Mean (SD); min - max** | **Mean (SD); min - max** | | **SMD^%^** | **p value** | | **p value** |
| Psychological distress | | 20.08 (6.44) | | 18.85 (6.43) | 1.22 | 0.12 | | 0.13 |
|  | | **n (% within year of data collection) Yes** | | **n (% within year of data collection) Yes** |  |  | |  |
| Suicidal ideation | | 13 (10.4) | | 11 (8.3) |  | 0.56 | | 0.51 |
| Help-seeking confidence | | 70 (54.7) | | 72 (54.1) |  | 0.93 | 0.79 | |
| Help-seeking behaviour | | 21 (17.1) | | 14 (10.5) |  | 0.13 | 0.34 | |

^$^ Adjusted for type of medical degree and presence of children at home.

^%^ Standardised Mean Difference
