# Supplementary material for: Effectiveness of a multi-modal hospital-wide doctor mental health and wellness intervention
Source: BMC Psychiatry. 2022 Apr 6;22:244. doi: 10.1186/s12888-022-03908-0 (PMC8983801; doi:10.1186/s12888-022-03908-0)
Supplement: Supplementary file 5 — Additional file 5: Table 5. The effect of a multi-modal doctor intervention on workplace factors (unadjusted and adjusted analyses) on consultants and fellows (n = 170). [file 12888_2022_3908_MOESM5_ESM.docx]

**Additional Table 5.** The effect of a multi-modal doctor intervention on workplace factors (unadjusted and adjusted analyses) on consultants and fellows (n = 170).

Mean (SD) values for each risk factor are shown before and after the intervention, with standardised mean differences (SMD) used to allow comparison of the effect sizes.

|  | **Unadjusted** | |  |  | **Adjusted^$^** |
| --- | --- | --- | --- | --- | --- |
|  | **Baseline (2017 sample)** | **Follow-up (2019 sample)** |  |  |  |
|  | **Mean (SD); min - max** | **Mean (SD); min - max** | **SMD^%^** | **p value** | **p value** |
| Hours worked/week | 44.83 (23.54) | 44.73 (13.69) | 0.10 | 0.97 | 0.84 |
| Job satisfaction | 3.64 (1.12) | 3.98 (0.88) | -0.34 | 0.04 | 0.15 |
| Overall stress | 10.92 (7.31) | 8.89 (4.82) | 2.04 | 0.13 | 0.31 |
| Support (administration) | 2.68 (1.19) | 2.93 (1.36) | -0.15 | 0.51 | 0.58 |
| Work-life balance | 2.95 (1.13) | 3.14 (1.15) | -0.19 | 0.33 | 0.19 |
| Excessive workload | 3.17 (0.96) | 3.28 (1.14) | -0.12 | 0.53 | 0.76 |
| Bullying | 3.25 (1.35) | 2.71 (1.26) | 0.55 | 0.16 | 0.07 |

^$^ Adjusted for type of medical degree and presence of children at home.

^%^ Standardised Mean Difference
