# Supplementary material for: Effectiveness of a multi-modal hospital-wide doctor mental health and wellness intervention
Source: BMC Psychiatry. 2022 Apr 6;22:244. doi: 10.1186/s12888-022-03908-0 (PMC8983801; doi:10.1186/s12888-022-03908-0)
Supplement: Supplementary file 6 — Additional file 6: Table 6. Comparisons of mental health and help-seeking outcomes (unadjusted and adjusted) before and after a multi-modal doctor intervention among consultants and fellows. [file 12888_2022_3908_MOESM6_ESM.docx]

**Additional Table 6.** Comparisons of mental health and help-seeking outcomes (unadjusted and adjusted) before and after a multi-modal doctor intervention among consultants and fellows.

|  | **Unadjusted** | | | |  |  | | **Adjusted^$^** |
| --- | --- | --- | --- | --- | --- | --- | --- | --- |
|  | | **Baseline (2017 sample)** | | **Follow-up (2019 sample)** |  |  | |  |
|  | | **Mean (SD); min - max** | **Mean (SD); min - max** | | **SMD^%^** | **p value** | | **p value** |
| Psychological distress | | 15.11 (4.60) | | 15.0 (6.18) | 0.07 | 0.95 | | 0.47 |
|  | | **n (% within year of data collection) Yes** | | **n (% within year of data collection) Yes** |  |  | |  |
| Suicidal ideation | | 6 (10.9) | | 3 (3.8) |  | 0.11 | | 0.075 |
| Help-seeking confidence | | 365 (62.5) | | 58 (73.4) |  | 0.18 | 0.22 | |
| Help-seeking behaviour | | 8 (14.3) | | 10 (12.7) |  | 0.78 | 0.81 | |

^$^ Adjusted for type of medical degree and presence of children at home.

^%^ Standardised Mean Difference
